# Prevalence of Symptomatic Nonstenotic Carotid Disease Using Simultaneous Non-Contrast Angiography and Intraplaque Hemorrhage Imaging for MR Screen Protocol

**DOI:** 10.3390/diagnostics12102321

**Published:** 2022-09-26

**Authors:** Chae Rin Lee, Jun Cheol Yang, Ui Yun Lee, Seung Bae Hwang, Gyung Ho Chung, Hyo Sung Kwak

**Affiliations:** 1 Medical School, Jeonbuk National University, Jeon-ju 54907, Korea; 2 Division of Mechanical Design Engineering, College of Engineering, Jeonbuk National University, Jeon-ju 54896, Korea; 3 Department of Radiology, Research Institute of Clinical Medicine of Jeonbuk National University, Biomedical Research Institute of Jeonbuk National University Hospital, Jeon-ju 54907, Korea

**Keywords:** atherosclerosis, carotid artery, stroke, magnetic resonance imaging

## Abstract

Background: To determine the prevalence of symptomatic nonstenotic carotid disease (SyNC) using simultaneous non-contrast angiography and intraplaque hemorrhage (SNAP) imaging for patients with acute stroke as an MR screen protocol and to assess imaging findings of carotid plaques. Patients and Methods: From May 2020 to October 2021, 2459 patients with suspected acute neurological symptoms were evaluated with brain diffusion-weighted imaging (DWI) and carotid SNAP imaging. We analyzed the degree of stenosis and intraplaque hemorrhage (IPH) using SNAP imaging. Prevalence of SyNC and risk factors for stroke in patients with SyNC were determined. We performed subgroup multivariate analysis between SyNC and other etiologies of stroke (non-SyNC). Results: Of 4608 carotid arteries in 2304 patients enrolled in this study, 454 (9.9%) plaques (both lesions in 128 patients) were found on SNAP imaging. Of these plaques, 353 (77.8%) showed stenosis of <50%. Of plaques with <50% stenosis, 47 (13.3%) patients had a territorial acute focal infarction. Seventeen (36.2%) were classified with embolic stroke of undetermined source (ESUS) and SyNC. High maximal wall thickness and carotid IPH were identified as influencing factors for SyNC. Conclusion: For patients with <50% stenosis and territorial infarction, SyNC is a relatively important source of stroke. Especially, high maximal wall thickness and carotid IPH are important risk factors for SyNC.

## 1. Introduction

Classifying acute stroke etiology is essential to prevent recurrence. The TOAST criteria are the most commonly used to categorize stroke etiology [1]. The American Heart Association recommends carotid revascularization only for patients with symptomatic carotid stenosis of more than 50%, while management for patients with less than 50% stenosis has not been clarified [2]. According to TOAST criteria, stroke classification is based on the degree of stenosis, while embolic stroke of undetermined source (ESUS) is a diagnosis of exclusion, which accounts for 17% (9% to 25%) of all ischemic strokes [3].

Patients with ESUS have a recurrence rate of 4.5% per year despite treatment with antithrombotic agents [3]. In addition, many other studies have demonstrated that plaque thickness, specific plaque features, and intraplaque hemorrhage can be independent risk factors for stroke in general and ESUS in particular, regardless of the degree of stenosis [4,5,6,7].

Recently, patients with territorial embolic infarction and carotid plaques <50%, called symptomatic nonstenotic carotid disease (SyNC), are classified as ESUS [8]. Therefore, in this study, we analyzed brain MR imaging, including simultaneous non-contrast angiography and intraplaque hemorrhage (SNAP) imaging, of consecutive patients with acute neurological symptoms. Our aim was to identify acute infarction patients that could be classified with SyNC and to analyze risk factors for SyNC and other stroke etiologies (non-SyNC) based on SNAP imaging.

## 2. Materials and Methods

This study was approved by our institutional review board (IRB), and informed consent was waived by our IRB due to its imaging analysis and retrospective design. 

### 2.1. Patients

From May 2020 to October 2021, we analyzed brain MRI with intracranial and carotid MR angiography of 2459 consecutive patients with acute neurological symptoms at our emergency room. During this period, all patients underwent initial brain MRI with MR angiography and a SNAP sequence for evaluation of carotid plaque to detect any neurological symptoms or signs, such as headache, dizziness, giddiness, or vertigo. The exclusion criteria were as follows: (1) poor MR imaging quality to evaluate vessel walls and plaques, (2) incomplete coverage of both carotid arteries on the SNAP sequence, and (3) young patients aged below 40 years to reduce selection bias. 

### 2.2. MR Imaging Protocol

MRI of conventional brain imaging and angiography was performed with a 3T MRI scanner (Achieva; Philips Medical Systems, Amsterdam, Netherlands) with a 16-channel head coil. We added the SNAP sequence for evaluation of IPH and wall thickness in the CA. SNAP imaging examined the coronal section from the carotid artery to the VBA. The parameters of the SNAP sequence have been described previously [7]. The image parameters were as follows: TR/TE/TI = 10/4.7/490 ms, FA = 11°, ETL = 98, FOV = 149 × 149 mm, matrix = 187 × 216, scan times = 3 m 30 s. The overall scan time of the MR examination was approximately 30–35 min. 

### 2.3. Clinical Data Assessment

Clinical data for patients with a carotid artery with less than 50% stenosis and focal territorial acute infarction, including TOAST classification, basic demographics, and risk factors for atherosclerosis, such as diabetes, hypertension, dyslipidemia, current smoking, and history of coronary disease, were recorded. Embolic stroke of undermined source (ESUS) was defined according to the criteria proposed by the Cryptogenic Stroke/ESUS International Working Group as a non-lacunar brain infarct in the absence of the following: (1) extracranial or intracranial atherosclerosis causing ≥50% luminal stenosis in arteries supplying the area of ischemia; (2) major-risk cardioembolic source; and (3) any other specific cause of stroke (e.g., arteritis, dissection, migraine/vasospasm, drug misuse) [9]. We divided patients into two groups, namely, ESUS (SyNC) versus other stroke causes (non-SyNC) in patients with carotid plaque below 50% stenosis and focal territorial acute infarction. SyNC was defined as ESUS plus carotid plaque below 50% stenosis. 

### 2.4. MR Imaging Analysis

MR images including SNAP sequences were retrospectively reviewed by two neuroradiologists (with 30 and 20 years of experience, respectively) blinded to the purpose of this study. Two neuroradiologists analyzed the imaging quality, degree of stenosis, presence of carotid plaques, IPH, and measurement of maximal wall thickness. 

They assessed image quality by consensus using a four-point scoring system (1, poor; 2, adequate; 3, good; 4, excellent). Images with a score of 1 were excluded from the final analysis. Disagreements regarding image quality were resolved by consensus. Carotid plaque was defined as ≥2 mm thickness of the vessel wall relative to image slices from beneath or above the focal wall. IPH of carotid plaques was defined as high-signal intensity within the plaques on the SNAP sequence. High signal intensity was defined as an area with >150% intensity of the signal of the temporalis muscle. The percentage of carotid stenosis was estimated on MR angiography using the NASCET criteria [10]. Maximal wall thickness was measured at the highest point of plaque on SNAP imaging.

A DWI positive signal was defined as a hyperintense signal on the DWI trace with an associated decreased signal on the apparent diffusion coefficient map corresponding to an acute ischemic event at the time of the scan. Acute territorial ischemic events were first classified based on distribution (ipsilateral ICA territory, ipsilateral basal ganglia, and posterior circulation). Only DWI-positive events in the ipsilateral ICA territory, excluding lacunar infarction, were identified as in the DWI-positive category.

### 2.5. Statistical Analysis

Statistical analysis was performed using MedCalc software, version 16.4.2 (MedCalc, Ostend, Belgium) and SPSS 23.0 for Windows (SPSS, IBM, Chicago, IL, USA). Pearson’s chi-squared test, Fisher’s exact test, Student’s *t*-test, and Wilcoxon rank-sum test were used for categorical or continuous variables, as considered appropriate. Univariate and multivariate analysis models were used to investigate the factors associated with ESUS and SyNC. To avoid variable selection caused by spurious correlations, only variables showing a potential association (*p* < 0.2) in univariate analysis were included in the multivariate logistic regression model. A two-sided *p*-value of <0.05 was considered statistically significant.

## 3. Results

During the study period, 2459 patients underwent brain MRI including the SNAP sequence for the evaluation of acute neurological symptoms. Of these patients, 85 were excluded from this study due to poor image quality, 5 due to incomplete coverage from both carotid arteries, and 65 due to young age below 40 years. Finally, 4608 carotid arteries of 2304 patients were analyzed in this study. 

**Figure 1:**
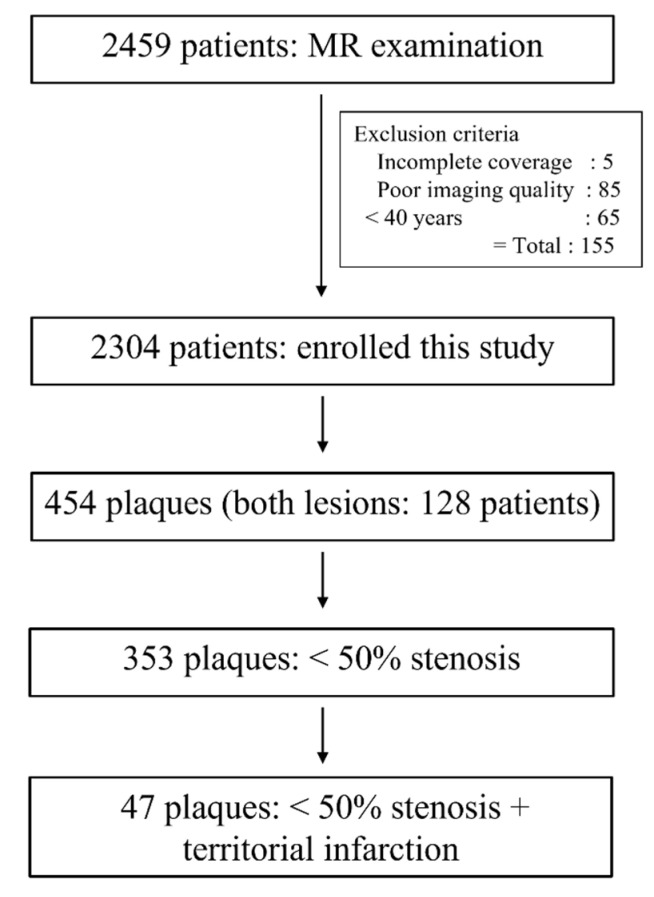
Flow diagram of patients.

Of the enrolled patients, 326 (14.1%) showed carotid plaques on SNAP imaging. Of these carotid arteries, 454 (9.9%) carotid plaques, including both lesions in 128 patients, were found. Of 454 carotid plaques, 353 (77.8%) had stenosis of <50%. Of carotid plaques with <50% stenosis, 47 (13.3%) showed a territorial acute focal infarction (Figure 1). Therefore, only 2.0% (47/2304) of enrolled patients and 1.1% (47/4608) of enrolled carotid arteries had <50% carotid stenosis and territorial acute focal infarction. 

Of 47 patients with <50% carotid stenosis and territorial acute focal infarction, 17 (36.2%) were classified with SyNC without the TOAST classification, and 30 were classified with non-SyNC because 10 had cardioembolic etiology, 14 had large artery atherosclerosis, and 6 had small vessel disease. Demographics and risk factors between SyNC and non-SyNC groups are shown in Table 1. Prevalence of previous stroke history in the non-SyNC group was significantly higher than that in the SyNC group (33.3% vs. 5.9%, *p* = 0.039).

**Figure 2:**
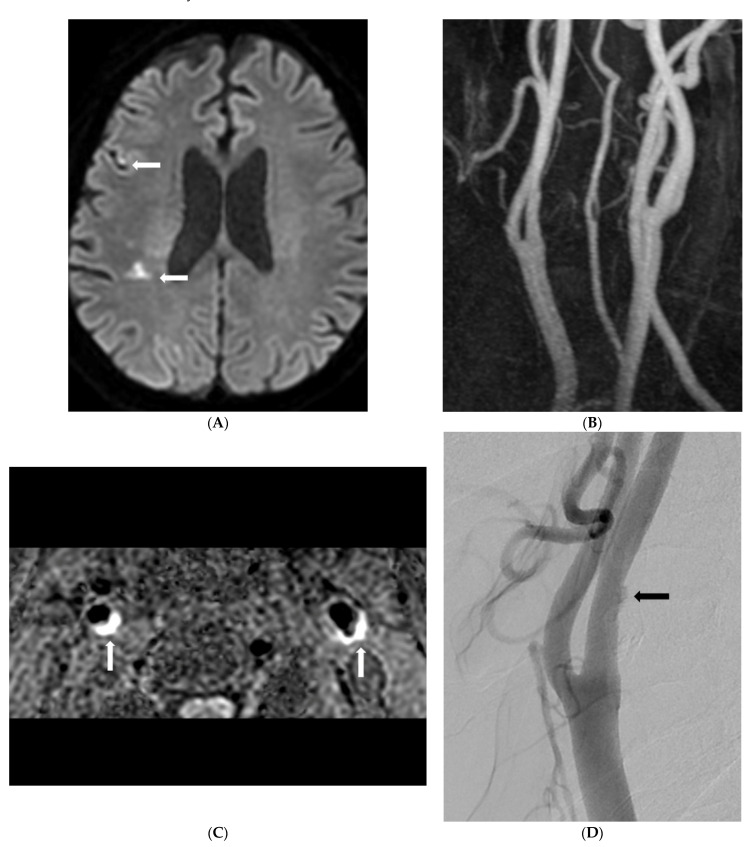

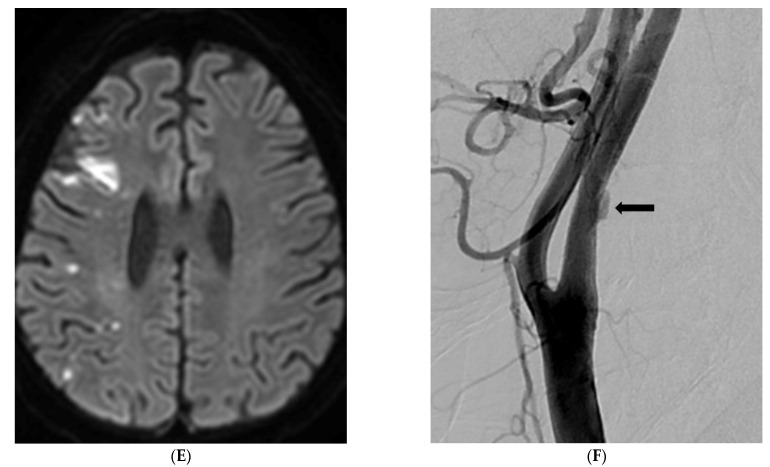
An 80-year old man with recurrent embolic stroke and symptomatic nonstenotic carotid disease. (**A**) Diffusion-weighted imaging shows diffusion restriction in right parietal white matter (arrows). (**B**) Carotid MR angiography shows mild stenosis of right proximal internal carotid artery. (**C**) SNAP imaging shows bilateral carotid intraplaque hemorrhage (arrows) and <50% carotid stenosis. (**D**) Carotid angiography shows focal ulceration of right carotid plaque (arrow). (**E**) Diffusion-weighted imaging shows multifocal and extended embolic infarction of right middle cerebral artery territory after one year. (**F**) Carotid angiography after recurrent embolic infarction showed increased ulceration and no interval change of stenosis (arrow).

Morphological analysis of carotid plaques on SNAP imaging between the two groups is shown in Table 2. The presence of carotid IPH in the SyNC group was significantly higher than that in the non-SyNC group (88.2% vs. 50.0, *p* = 0.009). Carotid stenosis and maximal wall thickness of carotid plaques were significantly higher in the SyNC group (Figure 2). 

Multivariate analysis was performed on factors associated with SyNC and ESUS (Table 3). Presence of carotid IPH (OR, 0.081 [0.01–0.672]; *p* = 0.02) and maximal wall thickness of carotid plaque (OR, 0.183 [0.034–0.978]; *p* = 0.047) were significantly associated with SyNC and ESUS.

## 4. Discussion

This study investigated the prevalence of SyNC or ESUS in patients with suspected neurological symptoms and signs. We found 14.1% of patients had carotid plaques in our screen MR protocol of patients with acute neurological symptoms. Additionally, 9.9% of carotid arteries showed plaque on SNAP imaging. Only 2.0% (47/2304) of enrolled patients had <50% carotid stenosis and territorial acute focal infarction. In addition, this study had two interesting findings. First, of 47 patients with <50% carotid stenosis and territorial acute focal infarction, 36% (17/47) with SyNC were classified with ESUS. Second, high maximal wall thickness and carotid IPH in patients with ESUS and SyNC were identified as influencing factors. 

The TOAST criteria, published in 1993, are the most commonly used criteria to classify stroke etiology [1]. Carotid artery plaques are considered a possible source of stroke according to TOAST but only if over 50% stenosis is present (large artery atherosclerosis, LAA). In addition, current American Heart/Stroke Association guidelines recommend management with carotid revascularization only in patients with symptomatic carotid stenosis of over 50% [11,12]. This recommendation is supported by information from the European Carotid Surgery Trial (ESCT) and North America Symptomatic Carotid Endarterectomy Trial (NASCET), which showed a significant reduction in future strokes after revascularization of symptomatic severe carotid stenosis but limited benefits in moderate stenosis [12,13]. Patients with carotid artery narrowing <50% meet diagnostic criteria for ESUS due to a peremptory view that carotid artery atherosclerosis with only mild stenosis generally does not have a pathogenic responsibility in acute embolic infarction [5,9]. Such diagnostic criteria ignore the fact that carotid artery atherosclerosis starts with a process of luminal plaque growth and wall remodeling during which a plaque is highly biologically active, despite not resulting yet in any stenosis [5].

ESUS with <50% stenosis is defined as a non-lacunar brain infarct without proximal arterial stenosis, another specific source of stroke, or a cardioembolic source [7,9,14]. ESUS reportedly accounts for 25% of ischemic strokes and accounts for nearly 300,000 cases annually in North America and Europe [5,9,15]. Moreover, numerous studies confirm that ESUS can play a potential role in stroke etiology through an association of certain plaque features that increase the risk of a cerebrovascular event, regardless of the degree of stenosis [16,17]. Some ESUS cases can be better classified as something else, such as cardioembolic stroke due to covert atrial fibrillation or as emboli due to patent foramen ovale [18,19,20]. Further, it is well-known that nonstenotic carotid plaque with unstable morphology and plaque components such as luminal irregularity, IPH, and plaque thickness, regardless of degree of stenosis, are much more common ipsilateral events of stroke in patients with ESUS [16,17].

The prevalence of carotid plaque in the screen MR study was clear. Our study analyzed the prevalence of carotid plaque using a screen MR protocol in patients with acute neurological symptoms. We found 14.1% of patients had carotid plaques in our screen MR protocol. Only 2.0% (47/2304) of enrolled patients had <50% carotid stenosis and territorial acute focal infarction. Therefore, the prevalence of SyNC with <50% carotid stenosis and territorial acute focal infarction in our MR screen protocol was extremely low.

Most studies about SyNC have been conducted on western people who have different features of carotid stenosis from Asians [21,22]. In this study, Asian patients with acute embolic stroke were evaluated in relation to SyNC by assessing imaging findings of carotid plaques. The result of our research was that of 47 patients with <50% stenosis and territorial acute focal infarction, 36.2% were classified with embolic stroke of ESUS and SyNC. In our study, SyNC was focused on IPH and maximal wall thickness using the SNAP sequence due to large screen MR examinations in patients with neurological symptoms. High maximal wall thickness and the presence of IPH were identified as higher in the SyNC group than in the non-SyNC group.

Recent studies have reported a relationship between the presence of IPH and the progression of acute ischemic stroke in both previously asymptomatic and symptomatic patients [6,23]. IPH is associated with plaque development and consequently causes luminal narrowing [23]. Altaf et al. [24] concluded that the highest event rate but also shortest time to the event was reported in patients with IPH and additional severe stenosis. Schindler et al. [6] found that patients with <50% stenosis have an annualized event rate of 9.0% with IPH versus 0.7% without IPH. Therefore, IPH may serve as a significant measure of risk for the development of future ipsilateral ischemic stroke [25]. In our study, the presence of carotid IPH in the SyNC group was significantly higher than that in the non-SyNC group (88.2% vs. 50.0%, *p* = 0.009). These findings suggested that SyNC might thus be an under-recognized stroke etiology in ESUS patients, and also that SyNC could be a potential cause of ischemic stroke. 

Other studies have demonstrated a significant association between maximum wall thickness and risk of acute infarction in patients with ESUS relative to the state of stenosis [26,27]. Zhao et al. [27] found that the maximum wall thickness was shown to be a stronger discriminator than luminal stenosis for HRP (AUC: 0.93 versus 0.81, *p* < 0.001). Ashley et al. [26] concluded that among ESUS cases, the total plaque thickness was greater on the ipsilateral side to the infarction than the contralateral, stroke-free side. Furthermore, one possible explanation for why maximum wall thickness had a stronger correlation with the plaque condition than luminal stenosis may involve positive remodeling of the plaque [28]. Positive remodeling involves the progression of an atherosclerotic plaque as it heads to the outward expansion of the outer wall border, while preserving the width of the lumen [28]. In our study, carotid stenosis and maximal wall thickness in the SyNC group were significantly higher than in the non-SyNC group. These findings suggested that non-stenotic plaque with maximum wall thickness could be an underlying mechanism of ESUS and also a reasonable target when screening for high-risk plaque.

We acknowledge this study has limitations. First, it is a retrospective study with an inherent possibility of selection bias. Second, we only analyzed IPH and wall thickness using SNAP imaging for SyNC. Carotid unstable plaque such as a large lipid-rich core and fibrous cap rupture could not be identified because we only used SNAP imaging of a large population. Third, this study may have a selection bias due to other potential sources of unrecognized atherosclerosis or cardioembolic emboli such as patent foramen ovale. Finally, our results are to be considered as evidence of an association between SyNC and ESUS, and not causation.

## 5. Conclusions

For patients with <50% stenosis and territorial infarction, SyNC is a relatively important source of stroke. Specifically, high maximal wall thickness and carotid IPH are important risk factors for SyNC. Further, high maximal wall thickness and carotid IPH were identified as influencing factors for SyNC. Future longitudinal studies on the association between SyNC and ESUS are warranted.

## Figures and Tables

**Table 1:** Demographic and risk factors between symptomatic nonstenotic carotid disease and non-symptomatic nonstenotic carotid disease groups.

	SyNC (*n* = 17)	Non-SyNC (*n* = 30)	*p*
Age, mean ± SD	74.5 ± 5.8	74.3 ± 10.0	0.933
Males, *n* (%)	13 (76.5)	17 (56.7)	0.175
Right plaques, *n* (%)	10 (58.8)	14 (46.7)	0.423
Hypertension, *n* (%)	7 (41.2)	19 (63.3)	0.142
Diabetes, *n* (%)	4 (23.5)	13 (43.3)	0.175
Hyperlipidemia, *n* (%)	5 (29.4)	7 (23.3)	0.733
Smoking, *n* (%)	8 (47.1)	8 (26.7)	0.156
Previous stroke, *n* (%)	1 (5.9)	10 (33.3)	0.039
Atrial fibrillation, *n* (%)	0 (0.0)	2 (6.7)	0.281
Alcohol, *n* (%)	4 (23.5)	3 (10.0)	0.235

Note: SyNC = symptomatic nonstenotic carotid disease; SD = standard deviation.

**Table 2:** Morphological analysis of carotid plaques between symptomatic nonstenotic carotid disease and non-symptomatic nonstenotic carotid disease groups.

	SyNC (*n* = 17)	Non-SyNC (*n* = 30)	*p*
Carotid IPH, *n* (%)	15 (88.2)	15 (50.0)	0.009
Carotid stenosis, median (IQR)	25 (10–40)	9.5 (0–25)	0.042
Maximal wall thickness, mean ± SD	4.5 ± 0.9	3.6 ± 0.8	0.001

Note: SyNC = symptomatic nonstenotic carotid disease; IQR = interquartile range; IPH = intraplaque hemorrhage; SD = standard deviation.

**Table 3:** Univariate and multivariable analysis associated with SyNC and ESUS.

	Crude OR	95% CIs	*p* Value	Adjusted OR	95% CI	*p* Value
Sex	2.485	0.655–9.427	0.181			
Hypertension	2.468	0.730–8.344	0.146			
Diabetes	2.485	0.655–9.427	0.181			
Smoking	0.409	0.117–1.428	0.161			
Previous stroke	8.0	0.925–69.218	0.059	15.19	0.974–0.978	0.052
Carotid IPH	0.133	0.026–0.687	0.016	0.081	0.01–0.672	0.02
Maximal wall thickness	0.229	0.084–0.624	0.004	0.183	0.034–0.978	0.047
Carotid stenosis	0.961	0.924–0.998	0.041			

Note: SyNC = symptomatic nonstenotic carotid disease; IQR = interquartile range; IPH = intraplaque hemorrhage; ESUS = embolic stroke of undetermined source; OR = odds ratio.

## Data Availability

The data presented in this study are available upon request from the corresponding author. The data are not publicly available due to privacy restrictions.
